# EEG classification of traumatic brain injury and stroke from a nonspecific population using neural networks

**DOI:** 10.1371/journal.pdig.0000282

**Published:** 2023-07-06

**Authors:** Michael Caiola, Avaneesh Babu, Meijun Ye

**Affiliations:** Division of Biomedical Physics, Office of Science and Engineering Laboratories, Center for Devices and Radiological Health, FDA, Silver Spring, Maryland, United States of America; National Yang Ming Chiao Tung University, TAIWAN

## Abstract

Traumatic Brain Injury (TBI) and stroke are devastating neurological conditions that affect hundreds of people daily. Unfortunately, detecting TBI and stroke without specific imaging techniques or access to a hospital often proves difficult. Our prior research used machine learning on electroencephalograms (EEGs) to select important features and to classify between normal, TBI, and stroke on an independent dataset from a public repository with an accuracy of 0.71. In this study, we expanded to explore whether featureless and deep learning models can provide better performance in distinguishing between TBI, stroke and normal EEGs by including more comprehensive data extraction tools to drastically increase the size of the training dataset. We compared the performance of models built upon selected features with Linear Discriminative Analysis and ReliefF with several featureless deep learning models. We achieved 0.85 area under the curve (AUC) of the receiver operating characteristic curve (ROC) using feature-based models, and 0.84 AUC with featureless models. In addition, we demonstrated that Gradient-weighted Class Activation Mapping (Grad-CAM) can provide insight into patient-specific EEG classification by highlighting problematic EEG segments during clinical review. Overall, our study suggests that machine learning and deep learning of EEG or its precomputed features can be a useful tool for TBI and stroke detection and classification. Although not surpassing the performance of feature-based models, featureless models reached similar levels without prior computation of a large feature set allowing for faster and cost-efficient deployment, analysis, and classification.

## Author summary

Traumatic Brain Injury (TBI) and stroke are devastating neurological conditions that affect hundreds of people daily. Unfortunately, detecting TBI and stroke without specific imaging techniques or access to a hospital often proves difficult and may lead to long-term health problems. An automatic portable biomarker can potentially facilitate patients triage and ensure timely medical intervention. Using a public dataset of electroencephalograms (EEGs) collected on a large variety of subjects, we were able to identify those as TBI, stroke, or normal with the use of natural language processing. We trained machine learning models with a large set of features calculated from each group of EEGs to classify between the different groups on unseen EEGs. These models classified between normal, TBI, and stroke with excellent performance, however, they required prior calculation of the features. Therefore, we trained a variety of different types of deep learning models that could work on little to no features. Overall, we showed these feature-based and featureless models performed similarly, and thus, allow for faster and cost-efficient deployment, analysis, and classification.

## Introduction

Traumatic brain injury (TBI) and stroke directly affect millions of people annually [1, 2]. In fact, the Centers for Disease Control and Prevention (CDC) estimates 176 Americans die from TBI-related injuries each day [3] and an American suffers a stroke every 40 seconds [2]. Treatment and recover options differ depending on the severity of TBI or stroke, however, without proper diagnosis or immediate detection, many cases go untreated.

The detection of TBI is challenging and usually achieved using one of two possible methods [1]: the Glasgow Coma Scale (GCS) [4], a clinical index universally used to classify TBI as mild, moderate, or severe, and CT scans to detect structural brain lesions. Though these methods are useful in the clinical management of TBI, they do not provide enough sensitivity to detect mild TBI or to monitor the progression of TBI [5]. The detection and assessment of stroke severity is traditionally performed using imaging and impairment tests such as the Medical Research Council Motor Assessment Scale [6]. However, these tests often do not include functional assessment of the patient [7]. There have been attempts to calculate such [7, 8] using tests such as the Modified Rankin Scale for global disability [9], the National Institutes of Health Stroke Scale [10] for neurological impairment, and the Barthel Index [11] for basic activities of daily life.

As new technologies and digital health tools are developed to satisfy the increasing need for alternative clinical assessment tools, there is a need to evaluate how well these new modalities capture the inherent features of TBI and stroke. One such non-invasive, easy-to-use, portable, and cost-effective modality is electroencephalography (EEG).

EEG has been investigated for assisting acute triage of TBI [12] and implementation of advanced EEG analysis methods has also demonstrated potential to differentiate severity of the TBI [13]. Stroke has also been evaluated using several EEG features including band power changes, brain symmetry index, and other spatiotemporal measures with varying results [14]. Recently, a push for more portable and characteristic EEG measures for stroke detection has been made [15–17], but a lack of external validation from a larger dataset is a limitation of such efforts. Although the potential of EEG-based efforts for TBI and stroke detection have been demonstrated in some studies, clinical applicability is still in debate [18–21]. With enough data, techniques such as machine learning may provide the ability to enhance the extraction of characteristic EEG features for TBI and stroke classification.

Our prior study used an independent dataset from a public repository and machine learning algorithm to select important spatiotemporal features and classify between normal, TBI, and stroke signals with an accuracy of 0.71 [22]. In addition, abnormal electrophysiological signals were observed without structural and biochemical changes following neural disruptive interventions, or even in the lack of apparent neurocognitive abnormality [23], suggesting that EEG has the potential to be a sensitive indicator of neuropathology. The results of several studies demonstrate that neural network-based models may be superior to other statistical models in EEG classification [24, 25]. Here we seek to improve classification performance by implementing a series of feature-based and featureless machine learning and deep learning models to obtain a significantly larger usable dataset. The feature-based models were built upon a diverse feature set consisting of statistical, spatiotemporal, and connectivity EEG measures to classify normal, TBI, and stroke patients. In addition, we evaluated the performance of a second set of deep learning models trained without the use of prescribed features which allowed the models to learn from ordinary EEG signals. Finally, we developed a tool to calculate patient-specific temporal features of importance from a classification prediction.

## Materials and methods

### Ethics statement

All patient data in the database were de-identified. Therefore, this study did not constitute human subjects research, and was exempted from Food and Drug Administration institutional review board review.

### Subjects

EEG data were obtained from the Temple University Hospital EEG Corpus (TUEG) v1.2.0 [26]. The TUEG contains 14,987 subjects with 26,846 deidentified clinical EEG sessions, totaling 69,652 EEG files (∼ 1,643 GBs), collected at Temple University from 2002-2017. For our study, subjects were selected through abbreviated patient records associated with each EEG file.

### Subject screening

**Fig 1:**
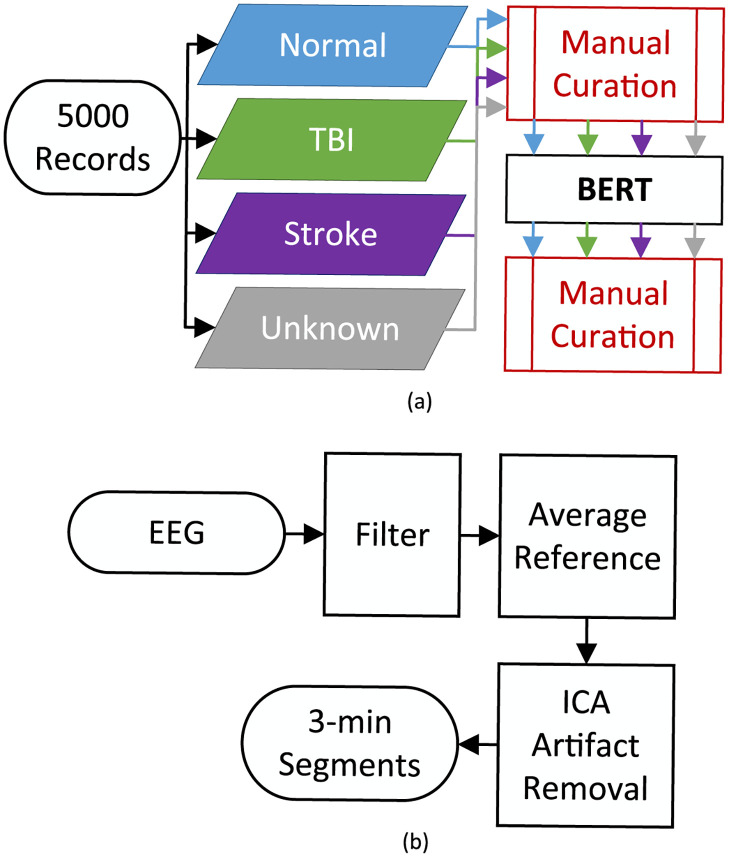
Data pipeline. The Data Extraction Flowchart (a) describes the steps taken to extract subjects from TUEG. First, a subset of records was manually classified as one of four categories and had keywords removed. Next, a BERT model was trained on this subset and used to classify the remaining records before a final manual curation. The Data Preprocessing Flow Chart (b) describes the preprocessing steps used on each extracted EEG: filtering, re-referencing and ICA artifact removal. The EEGs were then cut into 3-minute segments for analysis.

Subjects were selected as depicted in Fig 1a, including manual identification, Bidirectional Encoder Representations from Transformers (BERT) automatic selection, and final manual confirmation.

Each patient record was analyzed, and relevant text extracted using custom MATLAB scripts and workflows (Fig 1a). First, a subset of 5000 records were each manually read and categorized by a trained technician into one of four categories, “Normal”, “TBI”, “Stroke” or “Unknown”, using a custom MATLAB GUI. The grouping of “TBI” included traumatic brain injuries, and the grouping of “Stroke” included a history of stroke. For those that included both TBI and stroke or other neurological disease or conditions, including Dementia, Alzheimer’s Disease and epilepsy, “Unknown” was assigned. All other diseases or conditions, especially those who were noted as having “normal EEGs”, were categorized as “Normal”. We note that this distinction is different from that of a completely healthy patient.

This initial manual sorting was compared against two different previously published classifications from the TUEG, a keyword-based sort of Normal/TBI/Stroke records (denoted the Vivaldi Corpus) [22] and an automated sort of Normal/Abnormal EEGs (denoted Abnormal Corpus) [27]. Disagreements between our sort and the classifications made in the Vivaldi or the Abnormal Corpora were marked and reanalyzed by a second technician. Any records that we were not definitively categorizable were denoted as “Unknown”. As several subjects included multiple sessions or recordings, the possibility for an individual subject to be classified into more than one category was adjusted for by labeling any subjects with more than one category label as “Unknown”. After completion of the screening, we successfully labeled a total of 3938 subjects with 1,051 Normal, 328 TBI, 487 Stroke and 2,072 Unknown. With the treatment of data from multiple recordings as mentioned above, a final pool of 1,316 Normal recordings, 448 TBI recordings, and 614 Stroke recordings remained viable (see Table 1).

**Table 1:** Number of records/segments in each category during different stages of data extraction process.

Stages	Normal	TBI	Stroke
Initial Extraction	1316	448	614
BERT	4223	1614	3185
Manual Curating	2033	629	1146
Preprocessing	24028	6985	14469
Dimension Reduction	12009	3490	7229
Age and Sex Matching	3091	3351	2834

Note that the first three rows refer to individual recording files, while the last three rows refer to the 3-minute segments used for EEG classification.

Though, in some cases, multiple years had passed between repeated recording records, we felt it was best to include all possible recordings in this analysis. The mean time between recordings was 1.47 ± 2.51 years, 1.35 ± 2.50 years, and 0.7024 ± 2.24 years for Normal, TBI, and Stroke, respectively.

### BERT model

To label the remaining subjects, we utilized natural language processing (NLP) techniques by training a Bidirectional Encoder Representations from Transformers (BERT) base model pre-trained by Google on 800 million words from BooksCorpus and 2,500 million words from Wikipedia [28], on each record and associated classification in accordance with our aforementioned labeling.

To balance categories, a random selection of a dataset from the “Unknown” group was selected (800 records) based on the average record size of the other categories. A 20% holdout was selected from each category for model validation with the remaining 80% used for model training. The model was trained for 400 epochs with a resulting validation accuracy of 68.9% and a precision of 77.9% between Normal/TBI/Stroke categories.

To maximize the precision of the Normal/TBI/Stroke categories, a threshold was determined using a weighted precision score and all confidence scores, *s*, classified as “Unknown”. This conservative approach resulted in higher precision for each of the categories, albeit at the cost of data loss. The weighted precision, *w*_*p*_, was calculated such that:

$$w_{p}\left(s\right)=\frac{4p(s)+r(s)}{5}$$

where *p* was the precision and r was the proportion of true positives in the Normal/TBI/Stroke categories at score *s*. We found *w*_*p*_ to be highest at *s* = 0.81. Using this as our threshold, we were able to obtain a precision of 83.6% for the predicted Normal/TBI/Stroke classes.

The remaining data were then classified using our fine-tuned BERT model and precision thresholding procedures, resulting an additional 2,907 Normal records, 1166 TBI records, and 2571 Stroke records. This resulted in a final total of 4,223 Normal records, 1,614 TBI records, and 3,185 Stroke records (Table 1).

Using this new dataset, we once again removed any subjects with more than one category assigned to their recordings and conducted one more manual curation in which several targeted queries were reexamined. The first query checked for seizures and selected any records that contained the words “with [recurrent] seizure” with the bracketed word treated as an optional parameter. A second seizure query was conducted to select any remaining records with the word “seizure” but not the following phrases: “seizure-free”, “seizure free”, “no seizure”, or “if seizures are an important consideration”. In total, the seizure queries accounted for 3,417 records, most of which were then classified as “Unknown”.

Additionally, records that contained the phrases “evaluate for seizure” with “brain injury” as well as any that contained the phrases “evaluate for seizure versus stroke” or “evaluate seizure versus stroke” were reexamined. Finally, any records that contained “Alzheim” or “dement” were possible subjects with Alzheimer’s or dementia and were also excluded. We then excluded any recording where the subject was below 18 years old or older than 65, leaving 2,033 Normal records, 629 TBI records, and 1,146 Stroke records (Table 1).

### EEG preprocessing

Each of the 3,808 selected records was associated with an .EDF file containing a 19-contact EEG signal. EEG signals from different subjects were first standardized so that individual records conform with one another in terms of channels used and sampling frequency (fs). Similar to the methods used in Vivaldi et al. [22], EEG data were further pre-processed using MATLAB and EEGLAB (v.2022.0) [29] (Fig 1b). Signals (fs = 250 Hz) from 19 common channels in the standard 10-20 arrangement (FP1, FP2, F3, F4, C3, C4, P3, P4, O1, O2, F7, F8, T7, T8, P7, P8, FZ, CZ, PZ) were bandpass filtered from 1-100 Hz. Filtered signals were re-referenced to remove background noise by subtracting the average amplitude across all channels from each channel’s signal individually. EEG artifacts and non-brain function sources were removed using Independent Component Analysis (ICA) and ICLabel (v.1.3) [29] as described in Vivaldi et al. [22] and others [30–32]. Briefly, each independent component was labeled as either: brain, muscle, eye, heart, line noise, channel noise, or other. Then the independent components with non-brain function sources such as eye movements were excluded from signal reconstruction.

In most cases, a recording consisted of multiple continuous files. We combined these files into one continuous EEG and discarded the first minute to avoid any initial artifacts that may have occurred during recording. Following the methodology of Vivaldi et al. [22], this first minute removal was done for all combined files and then all records were split into 3-minute segments, with any partial segments discarded. The total number of segments in each category is depicted in Table 1, denoted as “preprocessing”.

### Calculating features

A set of 1406 features were calculated for each 3-minute EEG segment. These features include the 1330 features generated in [22] (phase-amplitude coupling [PAC] [33], absolute and relative power spectral density [PSD] within frequency bands, spectral entropy [SE], and inter-channel cross coherence [Coh]) as well as 76 channel-wise statistical values (mean, max, min, std). Frequency bands used were defined as 1–4 Hz (delta), 4–8 Hz (theta), 8–12 Hz (alpha), 12–16 Hz (mu), 16–20 Hz (beta), and 25–40 Hz (gamma).

### Data reduction

**Fig 2:**
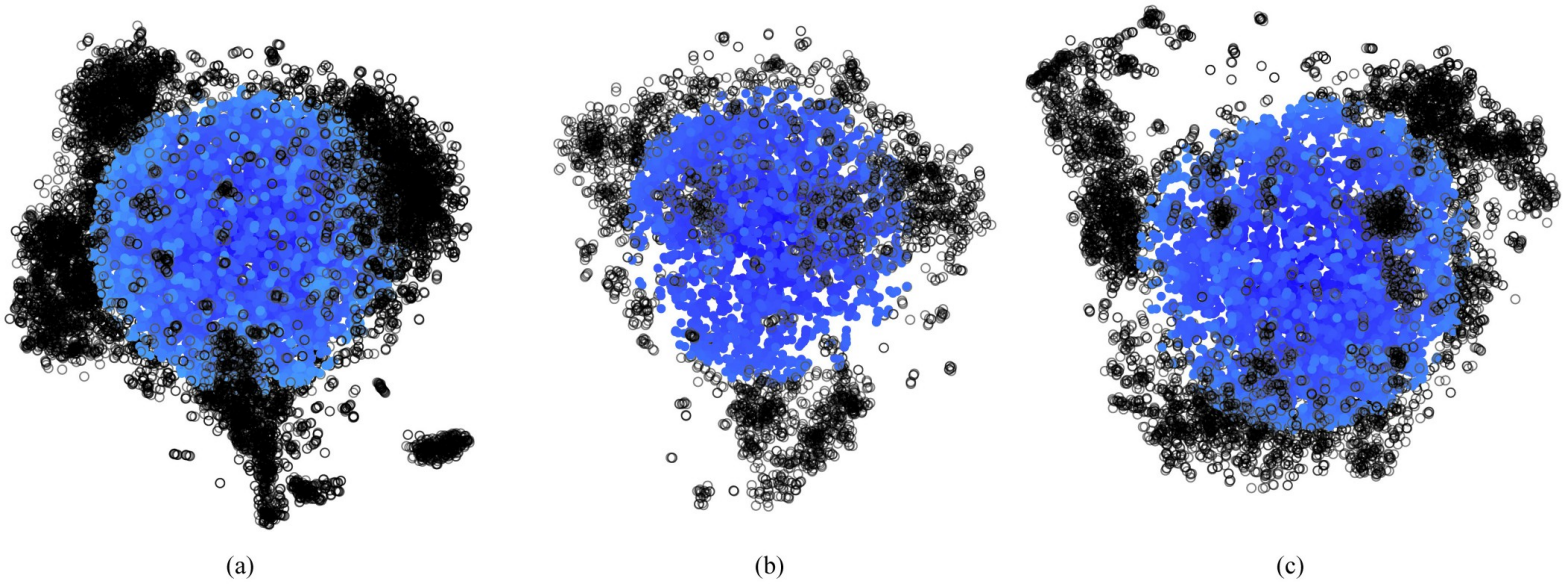
Dimension reduction of feature set for segment selection. Three-dimensional t-SNE of the Normal (a), TBI (b), and Stroke (c) segments. Each dot represents a single segment and is color coded based on its relative distance to all other segments (darker blue is closer). Black unfilled dots were excluded from all analysis and model training.

The TUEG may contain records where the patient had altered brain activity resulting from sleeping state or photic stimulation. We believe, however, that the majority of these segments are from awake or resting-state EEG recordings. To avoid these outliers in our 3-minute segments, a similarity analysis was conducted using t-Distributed Stochastic Neighbor Embedding (t-SNE) on each segment’s 1406 features (${\mathbb{R}}^{1406}\to {\mathbb{R}}^{3}$). This embedding plots similar segments close and dissimilar segments further away. Thus, we can calculate the total Euclidean distance from one point to each of its neighboring points and determine which segments are most similar to another. We excluded the furthest 50% of segments leaving the 50% most similar (in the 3D embedding of feature-space) segments. Each of the remaining segments were treated as independent in further analysis. These segments can be visualized in Fig 2 where the selected segments (blue) form an ellipsoid and the excluded segments (black) extend in different directions or branches. Overall, this similarity analysis led to a 50% reduction of segments, sacrificing only −10.55±2.40% of subjects per category.

Data were further reduced through age and sex matching to the resulting TBI population. That is, for each TBI subject, a subject in the Normal and Stroke categories were identified with the same sex and smallest difference in age (up to 5 years). Age and sex were automatically extracted from each patient record. When age was not found in the record, it was left blank unless the subject had another record or session where the age was recorded. In this case, the age was calculated using the time between recordings. Similarly, when sex was not noted, it was left blank.

**Fig 3:**
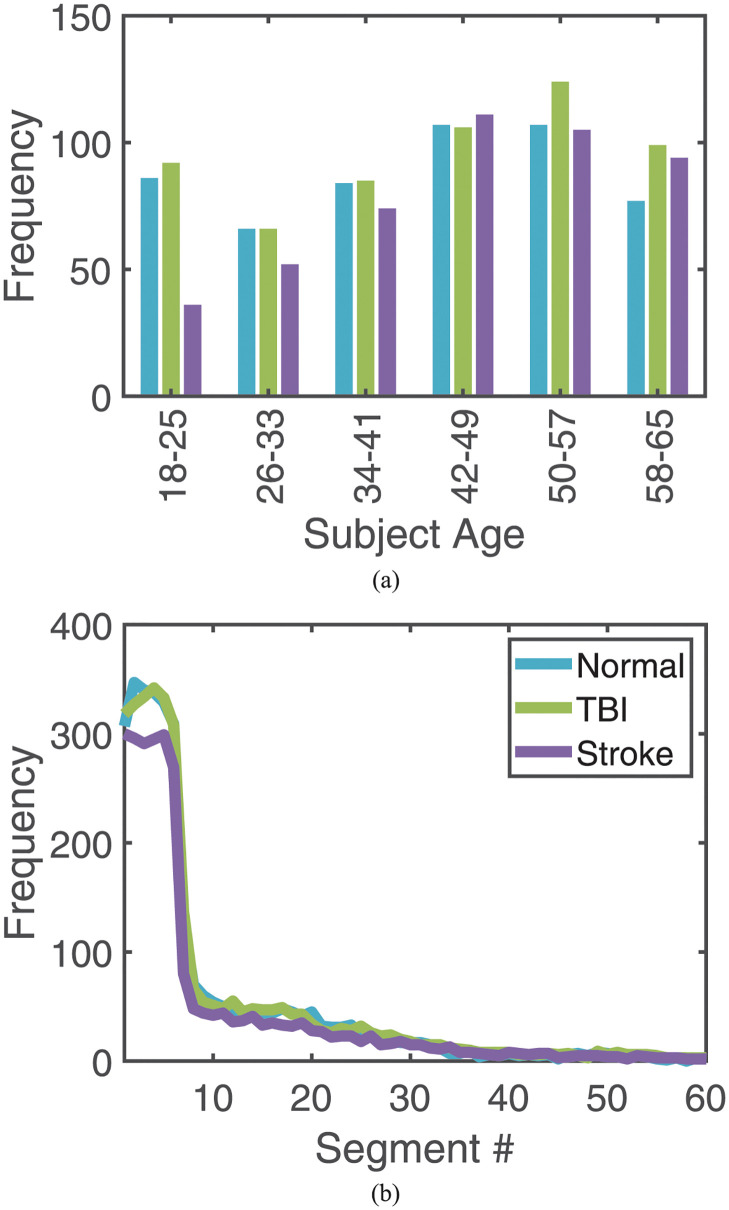
Training/Testing data summary. Age histogram of selected recordings between Normal, TBI, and Stroke categories (a). Number of segments for each category by segment number (b) where segment #*i* indicates the *i*th 3-minute segment of the EEG. The graph is truncated at 3 hours for visibility. Legend applies to both subfigures.

For each TBI recording, the matching algorithm first identified all Normal and Stroke segments for the same sex. Then, in that subset, the age from each recording was subtracted from that of the TBI recording, and its absolute value recorded. The minimum of these calculations was used to select matched subjects. In cases where there was more than one recording that satisfied this match, random selection was performed. The resulting age histogram is found in Fig 3a and the summary Table can be found in Table 2. Additionally, the number of segments used in training and testing is depicted in Fig 3b. Here segment numbers denote the segments temporal position in the EEG signal: segment #1 being the 3-minute segment from the 2nd minute to the 5th, segment #2 being the 3-minute segment from the 5th to the 8th and so on. We note that the frequency for all numbers greater than 60 was less than five but Fig 3b is only plotted to the segment #60 (3 hours) for visibility. The largest segment numbers used were 79, 350, and 474 for the Normal, TBI, and Stroke categories respectively.

**Table 2:** Summary of demographics for each EEG recording.

	Normal	TBI	Stroke
Subjects	498	471	421
# of Recordings	527	572	472
Age (yrs)	42.2 ± 13.4	43.2 ± 13.7	45.6 ± 12.2
Sex (M/F)	336/181	354/150	311/152

### Feature selection

To reduce the highly dimensional feature set, two methods were used for feature selection: linear discriminant analysis (LDA) [22] and the ReliefF algorithm [34]. LDA was optimized by selecting the best delta and gamma values using Bayesian optimization over a maximum of 30 objective function evaluations. Features with *δ* coefficient values above the mean of the *δ* values plus one standard deviation were selected. ReliefF was used to rank the importance of each feature and the top 100 were selected in this way.

### Classification models

To classify the EEG segments, a series of models were designed and trained on a subset of the data.

#### Feature-based models

The 1406 features as well as those selected by LDA or ReliefF were used to train a large net of machine learning models. These models included Decision Trees (fine, medium and coarse), SVMs (linear, quadratic, cubic, fine gaussian, medium gaussian, and coarse gaussian), and K-Nearest Neighbors (KNNs) (fine, medium, coarse, cosine, cubic, and weighted). Of those models tested, only cubic SVM and medium gaussian SVM were selected for further analysis. The cubic SVM consisted of a cubic kernel function with a heuristic subsuming procedure, a box constraint level of 1, and a one-to-one multiclass method. The medium gaussian SVM consisted of a Gaussian kernel function with scale 37, a box constraint level of 1, and a one-to-one multiclass method.

**Fig 4:**
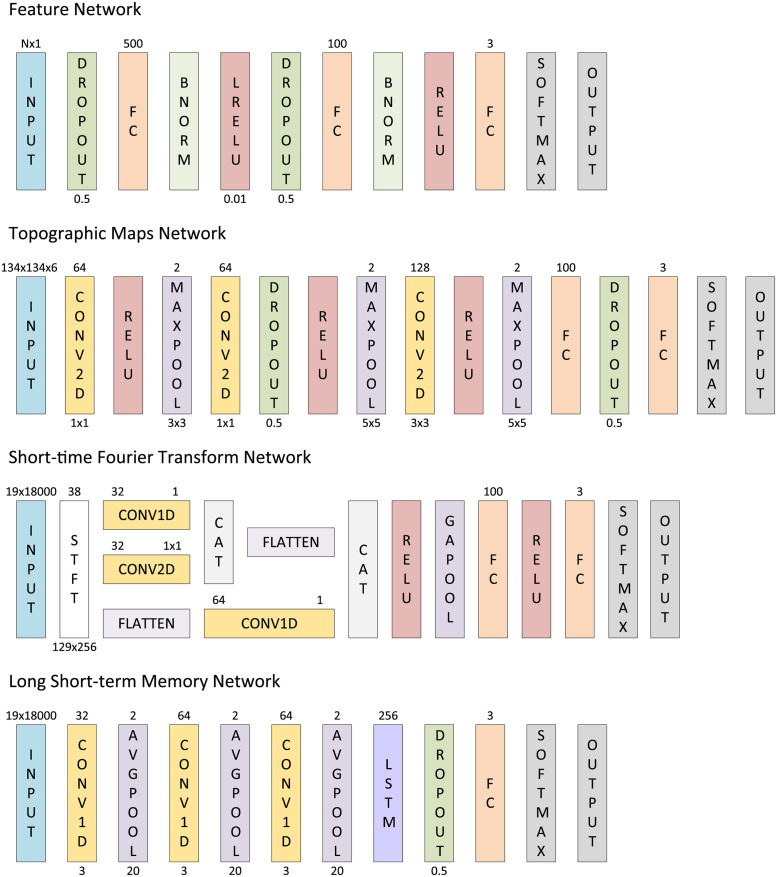
Network architecture for classification models. Each rectangle represents a layer in the network. Where appropriate the layer type is abbreviated (i.e., FC is a fully connected layer, BNORM is a batch normalization layer, RELU is a rectified linear unit, LRELU is a leaky RELU, convolutional layers are abbreviated as CONV and the dimension they operate on, STFT is a short-time Fourier transform layer, CAT is a concatenation layer, GAPOOL is a global average pool, AVGPOOL is an average pool, and LSTM is a long short-term memory layer). The number above the input displays the input size. The convolutional layers denote the number of filters above (left) and the kernel below (right). The fully connected and LSTM layers denote their hidden nodes above. The pool layers denote their stride above and their pool size below. Dropout and LRELU display their associated parameters below. STFT denotes its output channels above and its spatiotemporal output size below.

Additionally, a series of fully-connected deep neural nets, denoted Feature Networks, were trained (Fig 4). These Feature Networks take the feature vector as input and perform a 50% dropout before passing through a fully connected layer with 500 nodes, batch normalization, and a leaky rectified linear unit (ReLU) with scale 0.01. This set of layers was then repeated with 100 fully-connected nodes and a standard ReLU before a softmax and a cross-entropy loss classification.

These models were trained using a 10% holdout resulting in a 2782/3016/2551 training and 309/335/283 testing split for the Normal/TBI/Stroke categories. All data was standardized before training/testing.

The analysis was repeated using a smaller class of “early segments”, consisting of the first two 3-minute segments from each recording. This set of models used a 546/569/505 training and 60/63/56 testing split for the Normal/TBI/Stroke categories. Of those models tested, linear SVM with 196 LDA selected features and cosine KNN with 100 ReliefF selected features performed the best and were selected for further analysis. The linear SVM consisted of a linear kernel function with a heuristic subsuming procedure, a box constraint level of 1, and a one-to-one multiclass method. The cosine KNN consisted of a cosine distance function, and equal distance weight, using 10 nearest neighbors.

#### Featureless deep learning models

A series of deep learning models were also trained using a downsampled (100 Hz) EEG segment or associated measures as input instead of a feature vector. They are detailed below.

Topographic Map Network (TMN): Using a topographic map representing the change in relative PSD at each EEG channel location on the head and an interpolation across the scalp, we designed the Topographic Map Network (TNM) (Fig 4). The physical channel locations were calculated using a template boundary element model composed of three 3-D surfaces (skin, skull, cortex) extracted from the MNI (Montreal Neurological Institute) canonical template brain [35] provided by EEGLAB [29]. The TNM consisted of 16 layers and used 134 × 134 pixel topographic maps calculated over six powerbands (delta, theta, alpha, mu, beta, and gamma) as input. This 134(*S*) × 134(*S*) × 6(*C*) input underwent a 2D convolution (1 × 1 kernel) with 64 filters, a ReLU, a maxpool (3 × 3 kernel) with stride of 2, another 2D convolution (1 × 1 kernel) with 64 filters, a dropout layer (0.5 probability), a ReLU, a maxpool (5 × 5 kernel) with stride of 2, a convolution (3 × 3 kernel) with 128 filters, a ReLU, a maxpool (5 × 5 kernel) with stride of 2, a fully connected layer with 100 nodes, a dropout (0.5 probability), and a fully connected layer with 3 nodes. This output was then passed through a softmax layer and a cross-entropy loss classification was calculated.

Short-time Fourier Transform (STFT): Taking advantage of the spectral features of the EEG, we developed the Short-time Fourier Transform (STFT) Network with additional consideration of information in the time domain (Fig 4). The STFT Network used a 19 × 18000 sequence input and computed the one-sided STFT for each of the 19 channels. The real and imaginary STFT components were then concatenated into a 129(*S*) × 256(*T*) × 38(*C*) sequence. This sequence was then passed as input to both a 1D convolutional layer with 32 features (kernel = 1) and a 2D convolutional layer with 32 filters (kernel = 1 × 1) before being concatenated in the channel dimension and flattened. The STFT sequence was also flattened and passed through a 1D convolutional layer with 64 filters (kernel = 1) before being concatenated with the result of the flattened layer. This resulted in a 8320(*C*) × 559(*T*) sequence that then passed through the following series of layers: a ReLU, a global average pool, a 100 node fully connected layer, a ReLU, a 3-node fully connected layer, a softmax, and a classification output. Gradient-weighted Class Activation Mapping (Grad-CAM) [36] was later used with a moving average of 1 second to identify important elements of the signal.

Long Short-term Memory (LSTM): With a focus on temporal features of the signal, we developed a Long Short-term Memory (LSTM) network (Fig 4). The LSTM Network took a 19(*C*) × 18000(*T*) sequence as input and passed data through the following series of layers: a 1D convolution with 32 filters (kernel = 3), an average pool with stride of 2 (kernel = 20), a 1D convolution with 64 filters (kernel = 3), an average pool with stride of 2 (kernel = 20), a 1D convolution with 64 filters (kernel = 3), and an average pool with stride of 2 (kernel = 20). This output was then passed through an LSTM layer with 256 nodes, a dropout (0.5 probability), a fully connected layer with 3 nodes, a softmax, and a classification output layer.

**Fig 5:**
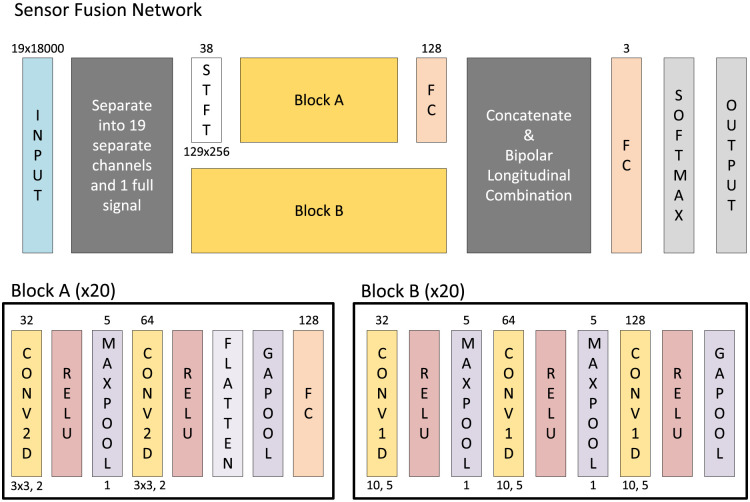
Deep learning architecture for the SFN. Top network displays main architecture of the model, but better readability, several groups of convolution and pool layers are grouped together in blocks (yellow) as well as some custom combination layers (grey). The blocks are denoted A and B and are reproduced below. Each block is done in parallel 20 times, one for each channel and one for a combination. Where appropriate the layer type is abbreviated (i.e., FC is a fully connected layer, RELU is a rectified linear unit, convolutional layers are abbreviated as CONV and the dimension they operate on, STFT is a short-time Fourier transform layer, and GAPOOL is a global average pool. The number above the input displays the input size. The fully connected layers denote the number of hidden nodes above. STFT denotes its output channels above and its spatiotemporal output size below.

Sensor Fusion Network (SFN): Finally, we attempted to combine the STFT spectral features with the topographic placement in the construction of a Sensor Fusion Network (SFN, see Fig 5). The SFN took a 19(*C*) × 18000(*T*) input and divided the signal into 20 distinct paths (one for each channel and one with the full signal) each with its own convolution and pool layers. Each path consisted of two subpaths, one calculated on the STFT and the other used original time series. The STFT subpath consisted of a 2D convolutional layer with 32 filters (3 × 3 kernel, 2 × 2 stride) followed by a ReLU, a maxpool (5 × 5 kernel), a convolutional layer with 64 filters (3 × 3 kernel, 2 × 2 stride), a ReLU, a flatten layer, a global average pool, and a 128 node fully connected layer. The other subpath consisted of a 1D convolutional layer with 32 filters (kernel = 10, stride = 5), a ReLU, a maxpool (kernel = 5), a 1D convolutional layer with 64 filters (kernel = 10, stride = 5), a ReLU, a maxpool (kernel = 5), a 1D convolutional layer with 128 filters (kernel = 10, stride = 5), a ReLU, and a global average pool. The output of both these subpaths were concatenated in the channel dimension. Each channel-specific output was then subtracted from its matching pair as described by the bipolar longitudinal pattern [37] and concatenated with the network output from the full signal. This output was then passed through a 3-node fully connected layer, a softmax, and a classification layer.

Each of these models were trained on 80% of the data with the remaining 20% used for independent validation using the ADAM optimizer with initial training rate 3 × 10^−4^. Training was manually stopped when loss began to stall or when 90 epochs was reached. In some cases, the model was trained again at a different initial learning rate to either speed up convergence or to avoid exponential loss growth. When the model underfit, techniques such as adding or removing layers, adjusting hyperparameters, and changing layer properties (e.g., kernel size, pool size, etc.) were adjusted to minimize loss. Overall, more than 30 models were trained before the network architectures described above were selected.

### Model performance

Model performance was evaluated with a Receiver Operating Characteristic (ROC) curve, calculated for each class in each model using the independent testing data. As ROC is mostly used as a binary comparison, the classes were compared in a one-versus-all coding design where one class would be the positive case and the remaining two would be considered the negative case. An average ROC curve was also calculated using the micro-average. Finally, the area under the curve (AUC) was calculated for each curve as a comparable metric of performance. For convenience, we refer to the AUC under the Normal ROC as nAUC, the AUC under the TBI ROC as tAUC, the AUC under the Stroke ROC as sAUC. In all other cases, the AUC reported for the model refers to the micro-average of the three classes.

### Computer hardware

All calculations were computed using on Dell laptops with 10th generation intel processors (i7 or i9) with 8 cores. When possible, deep learning was done utilizing an NVIDIA GeForce RTX 3070 laptop GPU with 8 GB of memory.

## Results

### Feature selection

**Fig 6:**
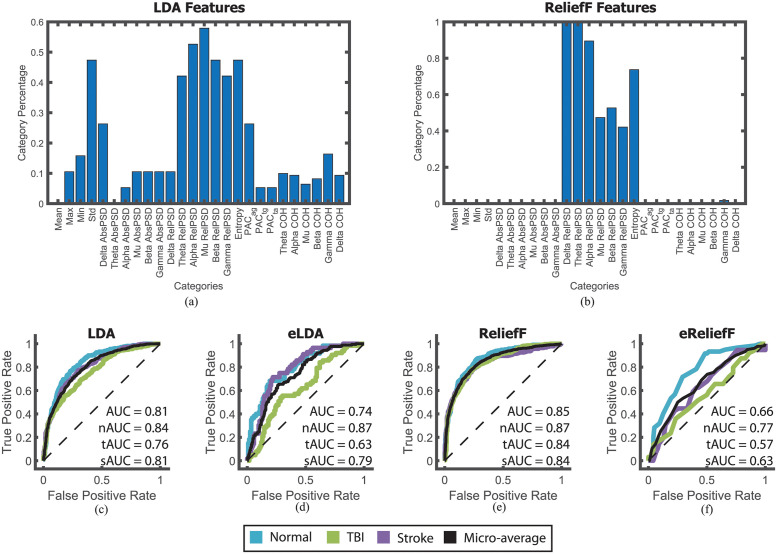
Feature selection and machine learning performance. LDA feature selection (a) selects 192 features with most being relative PSD band features and standard deviation category. ReliefF feature selection (b) selects the top 100 features after ranking with most being relative PSD band features. As most categories represent more than one feature, the category percentage shows the number of selected features in that category (e.g., LDA selected 2/19 channels in its feature selection giving it a category percentage of ∼ 0.1). These features were used to train two SVMs obtaining an AUC of 0.81 with LDA features (c) and an AUC of 0.85 with ReliefF features (e). A similar analysis was done using only the first and second segment of each recording. The resulting early LDA trained SVM (eLDA) is shown in (d) and the early ReliefF trained KNN (eReliefF) is shown in (f). The ROC curves are shown for each individual class (colored) and the micro-average (black). The black dotted line represents the ROC of a random classifier and is provided as a visual comparison.

A set of 1406 features were calculated from each EEG and two methods of feature selection were used to reduce the most important features: LDA and ReliefF. LDA was calculated using the full set of 1406 features and the entire set of chosen segments. Overall, LDA selected 192 features with the highest percentage of the features chosen in the delta, theta, alpha, mu, beta, and gamma relative PSDs, entropy, standard deviation, and delta absolute PSD (Fig 6a).

Next, the ReliefF algorithm ranked the importance of each the 1406 features, with the top 100 chosen. Fig 6b depicts the selected features in each category. ReliefF selected features primarily in relative PSD and entropy, sharing the many of the same features that LDA selected.

### Feature-based models

The resultant feature sets (full, LDA, and ReliefF) were used to train a series of machine learning models. These models included Decision Trees, SVMs, and KNNs. The best performing model using the LDA feature set was the medium gaussian SVM model with nAUC = 0.84, tAUC = 0.76, sAUC = 0.81, and AUC = 0.8054 (Fig 6c). The best performing model using the ReliefF feature set was the cubic SVM model with nAUC = 0.87, tAUC = 0.84, sAUC = 0.84, and AUC = 0.8516 (Fig 6e).

The machine learning models performed significantly worse when using the full feature set. The full feature set scored 6.8 (55.2% v. 62%) points lower in testing data accuracy when compared to the LDA medium gaussian SVM and 13.8 (56.2% v. 70%) points lower when compared to the ReliefF cubic SVM. This implies that the lower dimensionality of the LDA and ReliefF feature sets allowed for more optimized and quicker convergence, making them more practical and cost-effective.

In anticipation of the potential introduction of bias or additional noise into the system by including multiple 3-min segments from each subject, an additional analysis was performed to evaluate the accuracy of the early EEG segments. This was achieved by incorporating only the first and second segment of each EEG before performing feature selection. The best model trained on LDA features from early segments (eLDA) was a cubic SVM model with nAUC = 0.80, tAUC = 0.63, sAUC = 0.79, and AUC = 0.7419 (Fig 6d) while the best model trained on ReliefF features from early segments (eReliefF) was a KNN model with nAUC = 0.77, tAUC = 0.57, sAUC = 0.63, and AUC = 0.6588 (Fig 6f). Since eLDA and eReliefF were trained on roughly 20% of the data used for the other feature-based models, their low performance is most likely due to a lack of sufficient training data. Therefore, with the potential increase in training data and model performance of the other feature-based models, we can conclude that any additional noise or bias in the system is not detrimental to our classifications.

**Fig 7:**
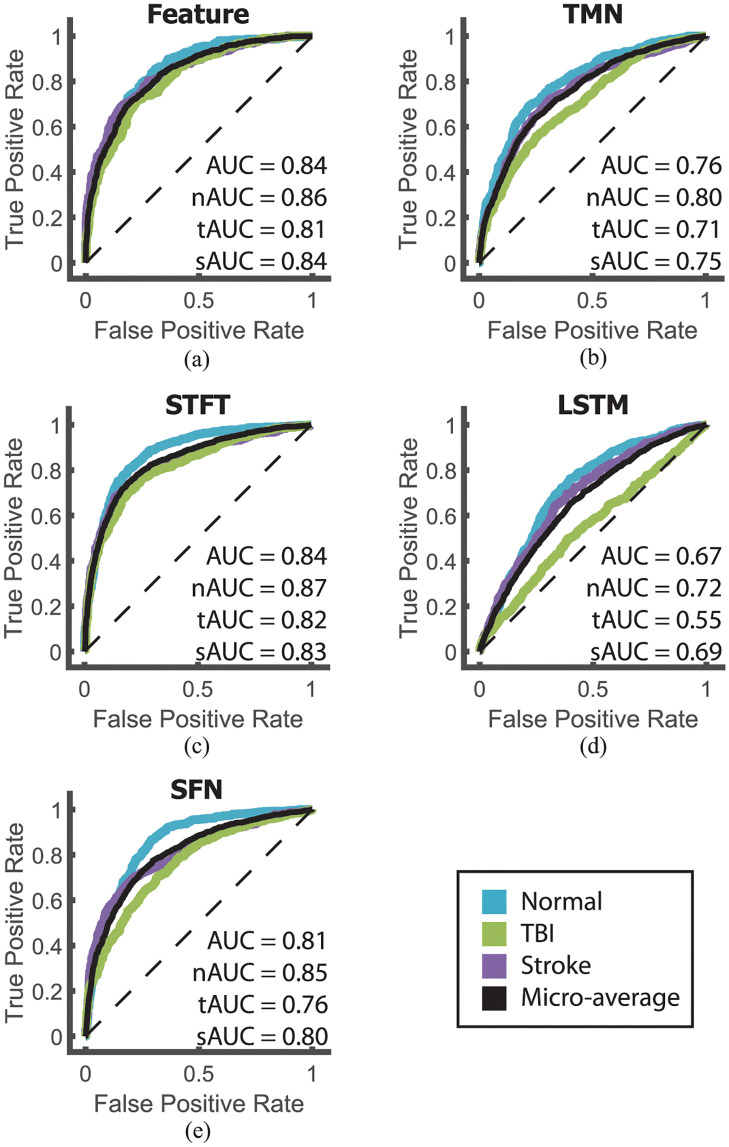
Deep learning model performance. ROC curves for the Feature Network (a), the Topographic Map Network (b), the Short-time Fourier Transform Network (c), The Long Short-term Memory Network (d), and the SFN (e). The ROC curves are shown for each individual class (colored) and the micro-average (black). The black dotted line represents the ROC of a random classifier and is provided as a visual comparison.

Finally, we tested whether a combination of our feature set and deep learning would improve the performance of SVMs mentioned above. The Feature Networks (Fig 4) were designed to allow for more complex combinations of the feature sets with the assumption that these extra combinations would allow for better model performance. However, when the ReliefF feature set was used to train the Feature Network, it performed very similarly to SVM model with AUC = 0.8350 (Fig 7a). This was an improvement over that of the full feature set, which was only able to obtain AUC = 0.7987.

### Featureless deep learning models

In addition to the feature selection methods presented above, several deep learning models were trained without calculation of the full 1406 feature set (Fig 4).

The TMNs were designed to take advantage of the high percentage of relative PSD features selected by LDA and ReliefF as well as incorporate the physical location of the EEG contacts. This was done by first converting the 19-contact EEG signal into six topographic maps representing relative PSD levels at delta, alpha, mu, beta, and gamma frequency bands. A standard convolutional neural net (CNN) was then used to determine which of the three categories the 6 maps belonged to. The TMN performed worse than the Feature Networks with AUC = 0.7551 (Fig 7b) even though it used the most selected features.

Focusing on the spatiotemporal components of the EEGs, the STFT Network consisted of 16 layers and took the EEG signal as input. The second layer transformed the 19 × 18000 EEG sequence into a 129 × 38 × 256 spatiotemporal sequence using the real and imaginary values of the STFT. The sequence was then used as input to three pathways of CNNs to extract temporal, spatial, and spatiotemporal features before being recombined and classified. The STFT Network performed better than that of the LDA SVM and very similar to the ReliefF SVM and Feature Networks with AUC = 0.8416 (Fig 7c).

We then developed the LSTM Network which focuses on temporal convolutions and used an LSTM layer to correlate temporal associations in the EEG signal. Unfortunately, this network (and several of its variations) failed to produce adequate results. Overall, this network performed the worst with AUC = 0.6629 (Fig 7d). Additionally, the TBI performance was just above random chance with tAUC = 0.55 leading us to believe that temporal dynamics alone may not be sufficient for EEG classification.

Finally, we developed the SFN (Fig 5) using individual STFT and CNN blocks for each EEG channel and a common bipolar montage (i.e., bipolar longitudinal pattern) [37]. Our goal was to incorporate both spatiotemporal and temporal features while still including the relative position of the EEG contacts. In this way, the SFN can be thought of a spiritual combination of the above networks. Overall, the SFN performed similar to that of the LDA SVM with AUC = 0.8067 (Fig 7e).

### Model interpretability

**Fig 8:**
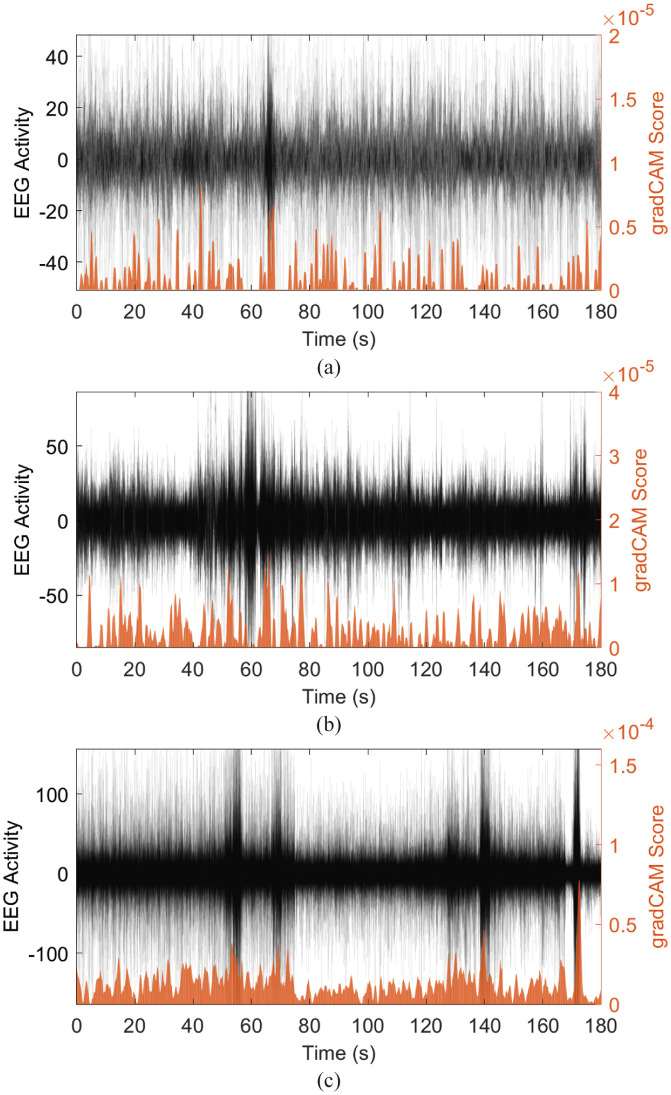
An example of highlighting important temporal components using Grad-CAM. An example of a 3-minute Normal (a), TBI (b) and Stroke (c) segment is shown. Each example displays all 19 contact EEG signals (overlapping black lines) and the Grad-CAM score (orange) at each time point. The Grad-CAM scores are displayed on the right y-axis with larger scores representing temporal components which strongly led to their respective prediction.

With a paucity of features given as input, many of the featureless deep learning models described above effectively operate as black boxes. Although this may be beneficial in cases, there is also benefit from insight into the model. For example, the STFT Network performs very similarly to that of the ReliefF SVM, however without prescribed features as input, it is hard to determine what architectural components are making the model choose a particular class. Since the STFT network is composed mostly of CNNs, we developed a tool using Grad-CAM [36] to determine which elements of our input had the most effect on our classification. This allows the user to determine which temporal segments of the EEG signal are indicative of the model’s prediction. Although this is a less generalizable biomarker, we can use this to help determine individual-level disease-specific features. An example of this tool for a correct Normal, Stroke, and TBI subject is illustrated in Fig 8.

## Discussion

### Database and model limitations

TUEG is a large public database that has been a common resource for those studying neurological diseases (e.g., TBI, seizures, and epilepsy) [22, 26] as well as other data mining initiatives [38]. The ability to extract information from large datasets is useful but also presents challenges in terms of selectivity. For example, since TUEG is not a disease-specific database, we only found ∼ 14% of the data useful for our application.

According to the CDC [39], there is a 1.4× higher incidence rate of TBIs for males compared to females. Furthermore, older adolescents and adults aged ≥ 65 years were more likely to visit the ER with a TBI. The TBI data extracted from TUEG shows similar trends with a higher proportion of male patients (nearly twice as many) and large number of patients in the 18–25 age group. However, the extracted TUEG data also includes many patients in the 42–57 age groups, differing from the CDC report. The source of this discrepancy could be twofold: we restricted our inclusion age range to 18–65 and are missing the necessary data to match these trends, or the types of scans included in the TUEG tend to include longer hospital stays than a typical ER visit. Additionally, the extracted data also indicated fewer young Stroke subjects, even after age and sex matching, consistent with reported Stroke incidence rates [40].

Maximizing the amount of training data available to a model typically allows for higher performance and more generality, thus we sought to extract the largest subset of data possible from the TUEG. However, this goal may have introduced additional error or bias. For example, our training data included multiple sessions from the same subject. Ideally, a model is trained on independent data segments, but due to the variability of EEG and duration between sessions (in some cases up to 16 years) we did not exclude any of these multiple sessions. Additionally, since each of these sessions yielded a new recording and associated patient record, we incorporated all records into our BERT model training and resulting predictions. This allowed the BERT model to learn on the largest number of records with the caveat that it also trained on multiple records from the same subject.

As each recording varied in length, we segmented each into multiple 3-minute segments before applying a similarity analysis. This analysis helped to account for factors that may alter brain activity (e.g., arousal status, stimulation, etc.), which were not denoted in the record. Unfortunately, there was no satisfactory way to fully determine if the EEGs were recorded in awake or sleeping subjects directly from the associated patient record. This issue can be mitigated as outlined in Vivaldi et al. [22] by only including early data from the recording at the expense of discarding a large amount of potentially good data. However, when we attempted to use this strategy, we found these models to underperform (Fig 6b and 6f). Alternatively, we assumed that each category consisted of similar awake data and excluded half the data based on feature space similarity. This excluded many potential and viable outliers that could possibly reduce the sensitivity of our model to real world scenarios.

Additionally, the subject session recording duration may be as little as 3-minutes or as much as 23.7 hours long. While subsequent segments may be similar, there is no guarantee that similarity holds from the first to the last segment of a recording. Without an a priori method to determine this likelihood, we treated each segment that passed our similarity analysis as independent. In this way, our model performed classifications between segments rather than subjects, and therefore the same subject may have segments in both training and testing sets. Although this may influence the overall accuracy of our model, the large number of segments in our testing set and the general variability of EEG keep our results generalizable. Nevertheless, we recommend testing on an independent or device-specific validation set before using the models presented herein for other applications.

### Model performance

Generally, each classification model calculates a score for every given datum and every class. This score is then compared to all other scores and a decision is made as to which category the item belongs. The final decision is normally controlled by a threshold that can be optimized to make a class as specific or sensitive as needed. We rated the performance of our models using ROC curves to demonstrate how the model changes with different thresholds. The resulting AUC calculations also allow for a single number comparison across different models. In general practice, successful models can be interpreted as “acceptable” when 0.7 ≤ AUC < 0.8, “excellent” when 0.8 ≤ AUC < 0.9, and “outstanding” above that [41]. However we note that these models would need to be clinically validated before an “acceptable” or “excellent” AUC is actually determined for one’s target population. As aforementioned, the goal of this project was to develop tools, models, and biomarkers that aid in the efforts to diagnose TBI and stroke from EEG segments, therefore, we focused on reporting AUC rather than optimizing our model towards a single metric (e.g., accuracy, sensitivity, specificity, etc.). Depending on one’s particular use case, an appropriate threshold can be chosen to optimize these metrics.

Overall, most of our trained models were considered “excellent”, with the exceptions of the “acceptable” TMN and the “unsuccessful” LSTM Network. It’s worth noting that all our models were trained on three classes equally, potentially decreasing the performance in any one class. The inclusion of both TBI and stroke was purposeful as we wanted to exclude the possibility of a global normal vs abnormal classifier. A similar technique was employed in Vivaldi et al. [22]. Additional factors that may have affected the performance of the models may be attributed to the inability to properly discern the history, severity, and recovery of each patient’s TBI or stroke. This could potentially lead to increased heterogeneity in each class and poorer classification.

One of the benefits of the feature-based models described above, is easy insight, that is, we can readily observe which features are key to the model’s success. Since only 100/1406 features were used to train our best preforming model (ReliefF SVM), its success is readily attributable to those 100 features. Additionally, since those features were developed a priori and are known measurements, we can include them as potential biomarkers towards these classifications. However, this approach limits us to the features we explicitly provide the model. The featureless deep learning models, on the other hand, use just the intrinsic properties found from their input data to make classification predictions. This is often more complex and could lead to more powerful models but with a lack of easily discernable user insight. Additionally, as they have already been trained on a large set of data, featureless models may benefit on the deployment side via very low computational cost (no feature calculation), speed, and portability of results derived directly from an EEG. In fact, the majority of our trained models are just around 100 MBs in size and do not need any prior data to make new predictions.

We developed a tool that provides some insight into the STFT model by using a technique called Grad-CAM. This allows us to observe how each data point in the input sequence contributes to the final model classification. In other CNNs, such as image classifiers, this technique may highlight a feature such as a dog’s ear shape as its main reason for classifying the image as “dog”. With an EEG sequence, however, this may be less intuitive, but it functions the same way: highlighting temporal components of the signal that were characteristic of its classification. We postulate this model insight to be important as it allows for follow-up clinical review on potentially troubling EEG segments, such as abnormal activity or even embedded artifacts.

The TMN model was found to be acceptable with AUC = 0.76, but in the TBI class resulted in tAUC = 0.71, making it barely acceptable. This is most likely because only relative PSDs and spatial information was given into the model, and it lacked some features such as entropy and Coh that ReliefF SVM provided. It is possible that with the addition of entropy and Coh features, the performance could be improved.

The LSTM Network consisted of a standard LSTM architecture and performed very poorly with a tAUC = 0.55. We speculate this poor performance was due to either inadequate volume of data or with respect to the temporal properties of data. Although we were able to approximate 3000 EEG segments, it is unclear whether that that was enough data to yield satisfactory results, especially with complex signals and complex neural nets. It is plausible that considerably more data is required to properly train a network of this architecture. Additionally, LSTMs are generally more expensive, limiting our ability to fine-tune the networks as with the other CNN models. Finally, another issue may be that all EEG segments were constructed uniform in length, allowing use some of the CNN techniques found in our networks, which is not strictly required for LSTM networks. At the cost of decreasing our EEG data by an order of magnitude, an attempt to train LSTMs without this prescribed uniformity might result in better performance due to temporal traits lost via our division.

Our largest model, the SFN was an attempt to isolate features from individual channels and their combinations. However, to create such a network, we needed 422 layers with 7.9 million learnable parameters. In comparison, the STFT Network only required 16 layers with 1.2 million learnable parameters. Even with this large increase in complexity, the SFN was able to achieve AUC = 0.81. We hypothesize that with a larger database, one could further improve the performance of the SFN. Alternatively, a synthetic data augmentation approach, using features found in each class, might prove advantageous, however, that was outside the scope of this study.

### Comparison to Vivaldi

While this study is directly comparable to the TBI and stroke model described in Vivaldi et al. [22], one major difference was in the construction of the classes. The Vivaldi Corpus was developed using simple keywords that did not discriminate on the context of the sentence. This was chosen as a conservative approach, but it also greatly reduced their class sizes, making techniques like deep learning infeasible. In contrast, in this study, use of a combination of manual reviewing, NLPs, and EEG segmenting, successfully increased the training set by up to 3178% and the testing set by up to 2004%, allowing for more sophisticated ML/AI techniques as well as more generality of our results. Additionally, we included changes in filtering procedures to exclude records for Alzheimer’s disease and dementia and to limit the age range to 18–65 years old. Finally, we yielded adequate data to enable age and sex matching to our TBI population, something that was not possible in Vivaldi et al., thus enabling our models to be more balanced and unbiased. Of note is that the Vivaldi model used TUEG v1.1 and that the version used in this study (v1.2) including data from 1437 more subjects.

Nevertheless, even with those changes, our study and Vivaldi et al. demonstrate similar results. Both selected features that consist of a high percentage of relative PSD. However, the Vivaldi model placed stronger emphasis on coherence features. As for performance, the LDA SVM model trained in the Vivaldi study had AUC = 0.8439, just slightly behind our ReliefF SVM (AUC = 0.8516). However, the Vivaldi SVM’s AUC was heavily influenced by its performance in stroke classification (sAUC = 0.91). The resulting nAUC = 0.79 and tAUC = 0.78 from the Vivaldi model, reduce its performance in classifying normal and TBI relative to the present model. Therefore, it may be speculated that a larger and more balanced training set than that used in Vivaldi et al. is needed to better generalize the changes associated with TBI and stroke. For a more detailed comparison between machine learning techniques and feature selection methods for TBI and stroke we point the reader to Vivaldi et al. [22].

## Conclusion

There is great interest in the academic community and by clinicians and device manufacturers in developing biomarkers and diagnostic tools for neurological disease. Our goal in this project was to develop tools, models, and biomarkers to enhance the utility of EEG in TBI and stroke diagnoses.

We demonstrated extraction of two meaningful disease-specific populations and a normal control group from the large nonspecific TUEG database with limited medical records using a BERT model fine-tuned on a subset of the data with high precision (0.84). To garner meaningful results from deep learning models, we increased the sample size of these populations by up to 432% using a dimension reduction and similarity comparison algorithm. With use of feature selection and machine learning techniques, we were able to use as little as 100 established features to identify these populations with AUC = 0.85. Five deep learning models that performed classification (with or without prescribed features) were also developed, each focusing on a different element of EEG time-series data. These models performed as well as, though not superior to, the machine learning (up to 0.01 AUC difference). Finally, we developed a tool using Grad-CAM to allow insight into the STFT Network to help determine patient-specific EEG features.

Overall, we conclude that machine learning models of EEG may be a viable tool for TBI and stroke detection and classification. Although not surpassing the performance of feature-based models, featureless deep learning models achieved very similar AUC without expensive computation associated with a large feature set. These featureless deep learning models allow for faster and more cost-efficient deployment, analysis, and classification, albeit at the expense of model interpretability. With proper fine-tuning and validation, we envision these tools leading to better clinical tests and diagnostic devices in the near future.

## References

[1] Centers for Disease Control and Prevention. Report to Congress on Traumatic Brain Injury in the United States: Epidemiology and Rehabilitation; 2015. 10.1016/j.apmr.2015.07.001 26184889

[2] Tsao CW, Aday AW, Almarzooq ZI, Alonso A, Beaton AZ, Bittencourt MS, et al. Heart disease and stroke statistics—2022 update: a report from the American Heart Association. Circulation. 2022;145(8):e153–e639. doi: 10.1161/CIR.0000000000001052 35078371

[3] Centers for Disease Control and Prevention. National Center for Health Statistics: Mortality data on CDC WONDER; 2022. Available from: https://wonder.cdc.gov/mcd.html.

[4] Teasdale G, Jennett B. Assessment of coma and impaired consciousness: a practical scale. The Lancet. 1974;304(7872):81–84. doi: 10.1016/S0140-6736(74)91639-0 4136544

[5] Jagoda AS, Bazarian JJ, Bruns JJ Jr, Cantrill SV, Gean AD, Howard PK, et al. Clinical policy: neuroimaging and decisionmaking in adult mild traumatic brain injury in the acute setting. Journal of Emergency Nursing. 2009;35(2):e5–e40. doi: 10.1016/j.jen.2008.12.010 19285163

[6] Carr JH, Shepherd RB, Nordholm L, Lynne D. Investigation of a new motor assessment scale for stroke patients. Physical therapy. 1985;65(2):175–180. doi: 10.1093/ptj/65.2.175 3969398

[7] Harrison JK, McArthur KS, Quinn TJ. Assessment scales in stroke: clinimetric and clinical considerations. Clinical Interventions in Aging. 2013;8:201–211. doi: 10.2147/CIA.S32405 23440256 PMC3578502

[8] Broderick JP, Adeoye O, Elm J. Evolution of the modified Rankin scale and its use in future stroke trials. Stroke. 2017;48(7):2007–2012. doi: 10.1161/STROKEAHA.117.017866 28626052 PMC5552200

[9] Quinn T, Dawson J, Walters M. Dr John Rankin; his life, legacy and the 50th anniversary of the Rankin Stroke Scale. Scottish medical journal. 2008;53(1):44–47. doi: 10.1258/RSMSMJ.53.1.44 18422210

[10] Lyden P, Lu M, Jackson C, Marler J, Kothari R, Brott T, et al. Underlying structure of the National Institutes of Health Stroke Scale: results of a factor analysis. Stroke. 1999;30(11):2347–2354. doi: 10.1161/01.STR.30.11.2347 10548669

[11] Mahoney FI, Barthel DW. Functional evaluation: the Barthel index. Maryland state medical journal. 1965;14(2):61–65. 14258950

[12] Hanley D, Prichep LS, Bazarian J, Huff JS, Naunheim R, Garrett J, et al. Emergency department triage of traumatic head injury using a brain electrical activity biomarker: a multisite prospective observational validation trial. Academic Emergency Medicine. 2017;24(5):617–627. doi: 10.1111/acem.13175 28177169

[13] Thatcher RW, North DM, Curtin RT, Walker RA, Biver CJ, Gomez JF, et al. An EEG severity index of traumatic brain injury. The Journal of neuropsychiatry and clinical neurosciences. 2001;13(1):77–87. doi: 10.1176/jnp.13.1.77 11207333

[14] Doerrfuss JI, Kilic T, Ahmadi M, Holtkamp M, Weber JE. Quantitative and Qualitative EEG as a Prediction Tool for Outcome and Complications in Acute Stroke Patients. Clinical EEG and Neuroscience. 2020;51(2):121–129. doi: 10.1177/1550059419875916 31533467

[15] Gottlibe M, Rosen O, Weller B, Mahagney A, Omar N, Khuri A, et al. Stroke identification using a portable EEG device—A pilot study. Neurophysiologie Clinique. 2020;50(1):21–25. doi: 10.1016/j.neucli.2019.12.004 32014371

[16] Wijaya SK, Badri C, Misbach J, Soemardi TP, Sutanno V. Electroencephalography (EEG) for Detecting Acute Ischemic Stroke. In: 2015 4th International Conference on Instrumentation, Communications, Information Technology, and Biomedical Engineering (ICICI-BME); 2015. p. 42–48.

[17] Cassidy JM, Wodeyar A, Wu J, Kaur K, Masuda AK, Srinivasan R, et al. Low-frequency oscillations are a biomarker of injury and recovery after stroke. Stroke. 2020;51(5):1442–1450. doi: 10.1161/STROKEAHA.120.028932 32299324 PMC7188582

[18] Schmitt S, Dichter MA. Electrophysiologic recordings in traumatic brain injury. Handbook of clinical neurology. 2015;127:319–339. doi: 10.1016/B978-0-444-52892-6.00021-0 25702226

[19] Rapp PE, Keyser DO, Albano A, Hernandez R, Gibson DB, Zambon RA, et al. Traumatic brain injury detection using electrophysiological methods. Frontiers in human neuroscience. 2015;9:11. doi: 10.3389/fnhum.2015.00011 25698950 PMC4316720

[20] Nuwer MR, Hovda DA, Schrader LM, Vespa PM. Routine and quantitative EEG in mild traumatic brain injury. Clinical Neurophysiology. 2005;116(9):2001–2025. doi: 10.1016/j.clinph.2005.05.008 16029958

[21] Arciniegas DB. Clinical electrophysiologic assessments and mild traumatic brain injury: state-of-the-science and implications for clinical practice. International Journal of Psychophysiology. 2011;82(1):41–52. doi: 10.1016/j.ijpsycho.2011.03.004 21419178

[22] Vivaldi N, Caiola M, Solarana K, Ye M. Evaluating performance of eeg data-driven machine learning for traumatic brain injury classification. IEEE Transactions on Biomedical Engineering. 2021;68(11):3205–3216. doi: 10.1109/TBME.2021.3062502 33635785 PMC9513823

[23] Munia TT, Haider A, Schneider C, Romanick M, Fazel-Rezai R. A novel EEG based spectral analysis of persistent brain function alteration in athletes with concussion history. Scientific reports. 2017;7(1):1–13. doi: 10.1038/s41598-017-17414-x 29222477 PMC5722818

[24] Lawhern VJ, Solon AJ, Waytowich NR, Gordon SM, Hung CP, Lance BJ. EEGNet: a compact convolutional neural network for EEG-based brain–computer interfaces. Journal of neural engineering. 2018;15(5):056013. doi: 10.1088/1741-2552/aace8c 29932424

[25] Guerrero MC, Parada JS, Espitia HE. EEG signal analysis using classification techniques: Logistic regression, artificial neural networks, support vector machines, and convolutional neural networks. Heliyon. 2021;7(6):e07858. doi: 10.1016/j.heliyon.2021.e07258 34159278 PMC8203713

[26] Obeid I, Picone J. The Temple University Hospital EEG Data Corpus. Frontiers in Neuroscience. 2016;10. doi: 10.3389/fnins.2016.00196 27242402 PMC4865520

[27] López S, Suarez G, Jungreis D, Obeid I, Picone J. Automated identification of abnormal adult EEGs. In: 2015 IEEE Signal Processing in Medicine and Biology Symposium (SPMB); 2015. p. 1–5. 10.1109/SPMB.2015.7405423 PMC4868184 27195311

[28] Devlin J, Chang MW, Lee K, Toutanova K. Bert: Pre-training of deep bidirectional transformers for language understanding. arXiv preprint arXiv:181004805. 2018;.

[29] Delorme A, Makeig S. EEGLAB: an open source toolbox for analysis of single-trial EEG dynamics including independent component analysis. Journal of neuroscience methods. 2004;134(1):9–21. doi: 10.1016/j.jneumeth.2003.10.009 15102499

[30] Jung TP, Makeig S, Humphries C, Lee TW, Mckeown MJ, Iragui V, et al. Removing electroencephalographic artifacts by blind source separation. Psychophysiology. 2000;37(2):163–178. doi: 10.1111/1469-8986.3720163 10731767

[31] Makeig S, Debener S, Onton J, Delorme A. Mining event-related brain dynamics. Trends in cognitive sciences. 2004;8(5):204–210. doi: 10.1016/j.tics.2004.03.008 15120678

[32] Delorme A, Jung TP, Sejnowski T, Makeig S. Improved rejection of artifacts from EEG data using high-order statistics and independent component analysis. Neuroimage. 2005;. 10.1016/j.neuroimage.2006.11.004 PMC2895624 17188898

[33] Tort AB, Komorowski R, Eichenbaum H, Kopell N. Measuring phase-amplitude coupling between neuronal oscillations of different frequencies. Journal of neurophysiology. 2010;104(2):1195–1210. doi: 10.1152/jn.00106.2010 20463205 PMC2941206

[34] Kononenko I, Šimec E, Robnik-Šikonja M. Overcoming the Myopia of Inductive Learning Algorithms with RELIEFF. Applied Intelligence. 1997;7:39–55. doi: 10.1023/A:1008280620621

[35] Oostenveld R, Praamstra P, Stegeman DF, van Oosterom A. Overlap of attention and movement-related activity in lateralized event-related brain potentials. Clinical Neurophysiology. 2001;112(3):477–84. doi: 10.1016/S1388-2457(01)00460-6 11222970

[36] Selvaraju RR, Cogswell M, Das A, Vedantam R, Parikh D, Batra D. Grad-CAM: Visual Explanations from Deep Networks via Gradient-based Localization. International Journal of Computer Vision. 2019;128(2):336–359. doi: 10.1007/s11263-019-01228-7

[37] Britton JW, Frey LC, Hopp JL, Korb P, Koubeissi MZ, Lievens WE, et al. Electroencephalography (EEG): An Introductory Text and Atlas of Normal and Abnormal Findings in Adults, Children, and Infants. Chicago: American Epilepsy Society; 2016. 27748095

[38] Nahmias DO, Civillico EF, Kontson KL. Deep learning and feature based medication classifications from EEG in a large clinical data set. Scientific Reports. 2020;10(1):1–11. doi: 10.1038/s41598-020-70569-y 32848165 PMC7450080

[39] Faul M, Wald MM, Xu L, Coronado VG. Traumatic brain injury in the United States: emergency department visits, hospitalizations, and deaths, 2002–2006. 2010;.

[40] Akyea RK, Vinogradova Y, Qureshi N, Patel RS, Kontopantelis E, Ntaios G, et al. Sex, Age, and Socioeconomic Differences in Nonfatal Stroke Incidence and Subsequent Major Adverse Outcomes. Stroke. 2021;52(2):396–405. doi: 10.1161/STROKEAHA.120.031659 33493066 PMC7834661

[41] Hosmer DW Jr, Lemeshow S, Sturdivant RX. Applied logistic regression. John Wiley & Sons; 2013.

